# Does Intensive Training of Attention Influence Cognitive Fatigability in Patients With Acquired Brain Injury?

**DOI:** 10.3389/fnins.2021.656876

**Published:** 2021-07-01

**Authors:** Anna Holmqvist, Aniko Bartfai, Gabriela Markovic, Marika C. Möller

**Affiliations:** ^1^Department of Rehabilitation Medicine, Danderyd University Hospital, Stockholm, Sweden; ^2^Department of Clinical Sciences, Karolinska Institutet, Stockholm, Sweden

**Keywords:** acquired brain injury, attention, cognitive fatigability, paced auditory serial addition test (PASAT), intraindividual variability

## Abstract

**Research Objectives:**

Impairments in attention and the speed of information processing are central to the experience of cognitive fatigue in patients with acquired brain injury (ABI). Attention may be improved through intensive training in a rehabilitation setting. The aim of the study was to investigate the feasibility of reducing cognitive fatigability (CF) using attention training and to explore the effect of two different approaches to attention training.

**Design:**

Randomised controlled study in a rehabilitation setting.

**Participants:**

59 patients (age 19–59 years) with mild to moderate stroke or traumatic brain injury in the early (<4 month) phase.

**Interventions:**

Patients were randomly assigned to intensive specific training with Attention Process Training (APT) or Activity-Based Attention Training (ABAT) for 3–5 days per week for a period of 5–6 weeks with a total of 20 h, in addition to traditional interdisciplinary rehabilitation.

**Main Outcome Measure:**

CF was conceptualised as performance decline in terms of an increased number of incorrect responses between the first and the last quintiles of the Paced Auditory Serial Addition Test (PASAT). A negative result was defined as fatigability. The evaluator of fatigability was blinded to treatment.

**Results:**

At baseline, there were no differences between the groups in age, education, reasoning, anxiety or depression. After training, a significant treatment effect was found (*p* = 0.020), as the APT-group, but not the ABAT-group, had improved. However, after controlling for baseline differences regarding CF on the PASAT-f, the difference was no longer significant.

**Conclusion:**

The results indicate that cognitive training might be a feasible method for reducing CF through attention training and that patients with high levels of CF benefit most from attention training. The type of intervention provided, whether specific or activity-based attention training, appears to be of less importance, as there was no treatment effect after controlling for the baseline level of CF. Future studies are required to confirm the validity of the findings.

## Introduction

Fatigue is one of the most prominent symptoms after brain injury. A substantial number of patients experience prolonged subjective problems that prevent them from returning to work and having an active leisure time (Olver et al., 1996; Glader et al., 2002), but treatment recommendations are still unsatisfactory and the evidence for different treatment approaches is weak (Wu et al., 2015).

There are two general approaches to treatment of the experience of fatigue – pharmacological treatment with methylphenidate (Johansson et al., 2017) and behavioural treatment, based on the assumption that mental fatigue is a condition reflecting an insufficient balance between the internal resources of mental energy and the ability to cope with the demands on the system that has been inflicted by cognitive impairments (Ashman et al., 2008). Thus, earlier behavioural studies attempted to decrease mental load through relaxation and mindfulness training (Johansson et al., 2012) or strengthen mental “capacity” using computerised working memory training (Björkdahl et al., 2013).

Inconclusive results from treatment studies could mirror the problem that fatigue is still poorly defined, and its measurement is limited by methodological and conceptual shortcomings (Kluger et al., 2013), as most studies rely on self-assessment questionnaires (DeLuca, 2005; Walker et al., 2019) that assess self-experienced mental fatigue. However, mental fatigue is a broad concept that does not capture the underlying causes of fatigue, nor is it precise enough to generate specific treatment hypotheses. Also, subjective ratings of fatigue are frequently influenced by other emotional states, such as depression (Arnold, 2008). These factors contribute to a low concordance between subjective self-assessed and objective performance-based fatigue measures and constitute major shortcomings in the evaluation of the effects of fatigue reducing interventions. Therefore, Kluger et al. (2013), have emphasised the importance distinguishing between subjective fatigue, as opposed to objectively measured fatigue.

Cognitive fatigue is a more stringently defined term that is used to show that mental fatigue is associated with thought-demanding tasks (Wylie and Flashman, 2017). To some extent, this term excludes the emotional fatigue that is common in depression (Wylie and Flashman, 2017) but the concept is not specific enough to be able to demonstrate that there is a *fatigability* associated with cognitively demanding tasks. One approach to create an even more narrowly objective assessment of fatigue is to conceptualise it as cognitive fatigability (CF), which is defined as a decline in performance on attention-demanding tasks by comparing performance at the beginning of a cognitively demanding test with performance at the end of the test (Kohl et al., 2009; Kluger et al., 2013), either in terms of a decrease in task accuracy (Walker et al., 2012; Morrow et al., 2015) or an increased response time (Berard et al., 2019). Also, increased intraindividual performance variability has been recommended as a metric for CF (Wang et al., 2014).

Holtzer et al. (2011) have successfully demonstrated that CF is triggered by tasks of executive attention, referring to the capacity to monitor and resolve conflicting information, which is subserved by the frontal cortico-striatal circuitry (Chaudhuri and Behan, 2000, 2004) as opposed to the alerting and orienting parts of attention (Holtzer et al., 2011). In line with this, previous studies (Lorist et al., 2005; Möller et al., 2014) have shown that the tests best suited to the assessment of CF are those which require controlled information processing or coordination of several cognitive domains.

Investigating the options for alleviation of CF in acquired brain injury is of particular interest, since treatment recommendations for CF are insufficient and the evidence for different treatment approaches is weak (Walker et al., 2019).

There are several systematic ways to strengthen the different aspects of information processing using systematic cognitive training (Cicerone et al., 2019). One of them is Attention Process Training, (APT). Attention Process Training is a theoretically anchored, evidence-based attention training method recommended after brain injury (Cicerone et al., 2019). APT includes targeted attention training based on hierarchical repetition to strengthen the attentional and executive functions at a functional level, but it also includes metacognitive aspects that promote a generalisation of strategies (Sohlberg and Mateer, 1987).

In a randomised controlled study (Bartfai et al., 2014) two methods to reduce the impact of attention dysfunction after acquired brain injury (ABI) were compared; a systematic cognitive training approach, Attention Process Training (APT), and Activity-Based Training of Attention (ABAT), focussing on adjustment and the use of strategies with the aim of improving occupational performance (Markovic, 2017).

Our group has previously demonstrated a performance decrement in attention-demanding tests, along with increased self-rated fatigue, in patients with mild traumatic brain injury (mTBI) (Möller et al., 2014). Furthermore, in an fMRI study (Möller et al., 2017) we have shown that mTBI patients did exhibit a decrease in performance on a psychomotor vigilance test (PVT) and an altered regional cerebral blood flow (rCBF) in several regions, including the left thalamus and superior frontal gyri, right precuneus and insula, together with the left/right medial frontal gyri and ACC, when compared to the healthy controls. Parts of these regions have been found to be active in tasks involving executive attention (Raz, 2004).

There is no gold standard for which test measures fatigability best, but the Paced Auditory Serial Addition Test (PASAT) is a multifactorial attention-demanding task measuring information processing speed, sustained attention, working memory and multitasking capacity (Gronwall, 1977) that has been used in several studies to capture CF in patients with multiple sclerosis (Walker et al., 2019), where fatigue is a major problem (Cantor, 2010). Though used in slightly different ways across studies as to interstimulus intervals (ISI) and cut off points for impairment, the performance on PASAT in the 3-second version of the test has shown a decline in MS-patients, based on the slope of correct responses throughout the test (Schwid et al., 2003) or by comparing the number of correct responses in the first and the last thirds of the test (Morrow et al., 2015).

Since CF has been associated with functional alterations in attentional networks in the brain (Möller et al., 2017) and since CF is, by its conceptual definition, closely associated with difficulties in sustaining attention and has been shown to be sensitive to executively demanding attention tasks (Holtzer et al., 2011), our hypothesis was that attention training could reduce CF after brain injury and that systematic attention training with metacognitive components (APT) might outperform ABAT by targeting the executive aspects of attention to a greater extent. Thus, the present study had two research aims: firstly, to investigate the feasibility of reducing CF using attention training and, secondly, to explore the effect of two different approaches to attention training. The present study is the first attempt to alleviate CF using systematic attention training.

## Materials and Methods

All of the data was collected from a large clinical trial investigating the effects of intensive cognitive rehabilitation of attention, and its impact on function and activity, after acquired brain injury. The specific details can be obtained from the study protocol (Bartfai et al., 2014).

### Participants

60 consecutive patients, 19–59 years, 40 men and 20 women in an early phase (<4 months) after mild to moderate stroke or traumatic brain injury with verified attentional impairment, were admitted to either inpatient or outpatient rehabilitation. The Glasgow Coma Scale (GCS) for the TBI patients was 13–15. The degree of stroke impairment at the point of impact was assessed based on medical journals in collaboration between a neuropsychologist and a rehabilitation medicine specialist. Patients included in the study were at a level corresponding to 13–15 GCS. The exclusion criteria indicate that patients with more severe cognitive impairment were not included. One of the patients did not complete the treatment, thus the final sample consisted of 59 subjects.

### Inclusion Criteria

Impairment in attention defined by the APT test (cut off scores of 70% or less on at least two of five subtests), scores in the lower average range and above for reasoning skills and abstract thinking (WAIS-III Matrix reasoning Scaled score ≥ 7) (Wechsler, 2003), age 18–60 years and a good understanding of Swedish. The presence of cognitive fatigability was not an inclusion criterion, since the data was collected from a clinical trial not focussing on CF (Bartfai et al., 2014).

### Exclusion Criteria

Moderate to severe aphasia, ongoing psychiatric illness, a history of anoxic episodes, substance abuse and severe pain. Severe memory impairment, neglect, an impaired visual field or motor impairment also led to exclusion. For more detailed information, see the previously published study protocol (Bartfai et al., 2014).

Patients who scored ≥ 10 in HADS (Zigmond and Snaith, 1983) were offered antidepressant treatment and were included three weeks after the initiation of pharmacological treatment, according to clinical praxis. These patients were reassessed before inclusion to ensure that their HADS scores met the inclusion criteria.

### Procedure

**FIGURE 1:**
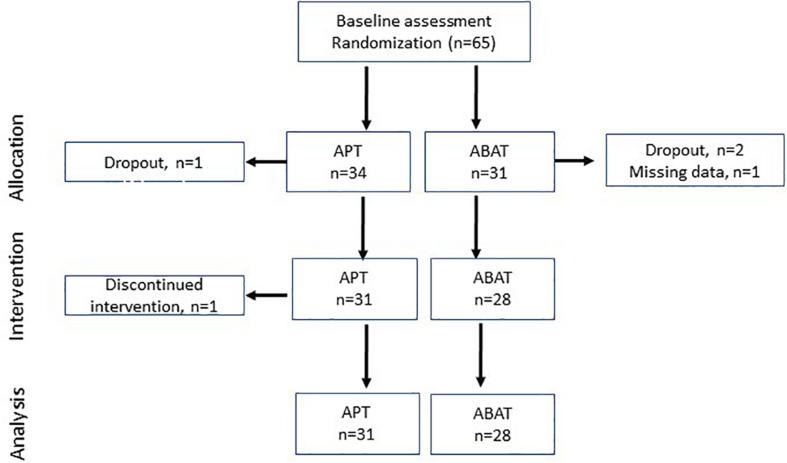
Flowchart of participants in each stage of the trial. APT, Attention Process Training; ABAT, Activity-Based Attention Training.

All of the patients were consecutively included in the study within the first four months of injury. They underwent an extensive neuropsychological assessment. In the present study, pre and post-intervention data (within two weeks before beginning the training and after the training) was used. The patients participated in an interdisciplinary brain injury rehabilitation programme (in and outpatient care) with an added 20 h of attention training, either APT (*n* = 31) or ABAT (n = 28), based on randomisation (Figure 1). The intensity of the training was 45–90 min, 2–3 times per week for 5–6 weeks. Since rehabilitation cannot be blinded, neither patients nor therapists were blinded to the intervention. However, the assessment was blinded as to the form of treatment (Bartfai et al., 2014).

### Assessment

#### Paced Auditory Serial Addition Test

The PASAT is a cognitively demanding test measuring mental processing speed and various aspects of attention and working memory functions (Gronwall, 1977). We conceptualised CF as a performance decline in terms of an increased number of incorrect responses between the first and the last quintiles of PASAT. The partitioning into quintiles, formerly applied in studies where cognitive fatigability was assessed with psychomotor vigilance tasks (Möller et al., 2017; Berard et al., 2019), was chosen with the intent to optimise the sensitivity to performance decrement.

The Swedish version of the test includes 60 pre-recorded numbers at a standardised pace of 2.4 s between numbers. The task is to sum each new number with the previous one and provide the correct answer before the next number is given. Higher scores indicate better performance. Performance was evaluated according to the manual (Gronwall, 1977).

Cognitive fatigability was measured as declining performance in terms of an increased number of incorrect responses on PASAT (2.4-second version); PASAT fatigability (PASAT-f). The PASAT-f was used as the primary outcome measure.

PASAT fatigability (PASAT-f) was calculated as follows: the material was divided into five sections of 12 numbers each, where the number of correct answers in the first section was subtracted from the number of correct answers in the last section. Fatigability was defined as a lower result at the end of the test compared to the beginning, which gives a negative value, and was reported as the percentage of correct answers in the first quintile [(number of correct answers in the first quintile/total number of correct answers for the entire task) ^∗^ 100] subtracted from the percentage of correct answers in the last quintile [(number of correct answers in the last quintile/total number of correct answers for the entire task) ^∗^ 100]. For example: [(4/40)^∗^100] – [(10/40) ^∗^ 100] = –15%. Intraindividual variability was estimated as the standard deviation of the number of correct responses for each quintile.

#### Ruff 2 & 7

The Ruff 2 & 7 Selective Attention Test (Ruff and Allen, 1996) is a continuous performance test that measures cognitive speed and sustained and selective attention. In this study, the test was used to investigate the correlation between visual attention as measured with Ruff 2 & 7 and fatigability. The Ruff 2 & 7 consists of twenty, fifteen-second trials, where the task is to identify and cancel the target digits 2 and 7. The digits are embedded among distractors. The distraction consists of alphabetical letters (automatic selective attention) and other numbers (controlled selective attention) for ten trials each. Sustained attention is measured as the total number of correctly identified targets. Higher scores indicate better performance. Performance was evaluated according to the manual (Ruff and Allen, 1996).

#### Digit Span

Verbal attention span and working memory was assessed with Digit span forward according to the Wechsler Adult Intelligence Scale procedures (Wechsler, 2003). The test was used to investigate the relation between attention span, unrelated to processing speed, and fatigability. The participant is asked to repeat a series of numbers in order of length, (between 2 and 9 numbers), two trials per length.

#### Hospital Anxiety and Depression Scale

Hospital Anxiety and Depression Scale (HADS) (Zigmond and Snaith, 1983) is a self-assessment questionnaire that was used to control for the effects of anxiety and depression, pre and post-intervention. The questionnaire consists of 14 items divided into the two subcategories of anxiety and depression. A score > 10 on either subscale indicates pathology.

### Interventions

#### Attention Process Training

Attention Process Training (APT) (Sohlberg and Mateer, 1987) is an intensive, function-specific and individualised cognitive training method targeting five attention levels; focussed, sustained, selective, divided, and alternating attention. The training program is comprised of structured visual and auditory exercises administered in a hierarchical manner, supplemented by metacognitive training, education about ABI related attentional deficits and training in generalising acquired strategies into daily life. The APT programme includes a screening instrument to assess attention dysfunction. The result of the test indicates the type and number of attention problems at hand and the suitable starting level for the training program. The APT was performed by a neuropsychologist.

#### Activity-Based Attention Training

Activity-Based Attention Training (ABAT) consists of standard occupational training that focusses on activity limitations due to attention dysfunction (Markovic, 2017). Activity-Based Attention Training was considered to be treatment as usual. The training includes compensatory strategy training in attention-demanding tasks in the domain of ADL, computerised tasks and group activities. The aim of the training is to improve occupational performance by building on adjustment and the use of these strategies. Examples of the compensatory strategies generally used were taking frequent breaks, using notebooks and verbal self-guidance. Activity-Based Attention Training was performed by an occupational therapist, either individually or in a group depending on the aim.

### Statistics/Data Analysis

Variables were summarised using standard descriptive statistical methods. The difference between pre-training and post-training (d-values) was calculated for the neuropsychological outcome measures that were administered pre-treatment and post-treatment. As to inferential statistics, non-parametric methods were used for variables that were not normally distributed. For continuous non-parametric data, the Mann-Whitney *U* test was used for comparison between groups and the Wilcoxon rank sum test was used for comparison within groups. For group comparisons based on categorical data, the Chi2 method was applied. Parametric methods were used for normally distributed data on the interval level. For independent samples, a *t*-test was used to compare treatment groups and a paired samples *t*-test was used for comparison within treatment groups. Depending on the data type, either a Pearson correlation or a Spearman’s rank was used for analysis of the associations between variables. To control for baseline differences, a univariate analysis of covariance was performed with d-values of fatigability as the dependent variable, group as the fixed factor and baseline fatigability as a covariate.

The significance level was set to *p* < 0.05 (2-tailed). Power was set at 0.85 (Bartfai et al., 2014). Data was analysed in IBM SPSS, version 23.

## Results

### Demographics

At baseline there were no differences in age, education, reasoning, digit span, anxiety/depression, type of injury or latency. In the APT group there was a trend toward more women than in the ABAT group (*p* = *0.054*) (Table 1). However, fatigability rates were comparable between males and females (*t* = *–*0.181, *df* = 57 *p* = *0.857).*

**TABLE 1:** Demographics and inclusion data for the Attention Process Training group (APT) and the Activity-Based Attention Training group (ABAT).

	APT	ABAT
	*N* = 31	*N* = 28
Age years, mean (SD)	45.2 (11.8)	43.9 (11.2)
Gender, n (% females)	14 (45%)	6 (21%)
Education		
• Elementary $\underline{<}$ 9 years, n (% participants)	1 (3%)	0
• High school (% participants)	7 (23%)	8 (29%)
• University < 4 years, n (% participants)	15 (48%)	14 (50%)
• University > 4 years, n (% participants)	8 (26%)	6 (21%)
Type of injury		
• Stroke, n (% participants)	26 (84%)	20 (71%)
• TBI, n (% participants)	5 (16%)	8 (29%)
Latency days, mean (SD)	60.1 (25.0)	58.8 (27.9)
Digit span forward	5.8 (1.1)	5.9 (1.3)
HADS-Anxiety, median (range)	5 (0–16)	3 (0–18)
HADS-Depression, median (range)	3 (0–15)	3 (0–15)

*HADS, Hospital Anxiety and Depression Scale; TBI, Traumatic Brian Injury.*

### Baseline Descriptive Data

At baseline, the APT group showed significantly more fatigability (PASAT- f) than the ABAT group, as well as a higher degree of performance variability. There were no significant baseline differences between the groups on PASAT total, Ruff 2 & 7 ADS or Ruff 2 & 7 CSS (Table 2). The baseline results on Ruff 2 & 7 ADS and CSS for the total group were in the lower normal T-score range (*M* = 45, *SD* = 10.53; *M* = 41, *SD* = 9.93). Thus, for further baseline statistics the two groups were merged.

**TABLE 2:** Values pre-training and post-training for the Attention Process Training group (APT) and the Activity-Based Attention Training group (ABAT) on neuropsychological measurements.

Measurements	Pre-training	Between group p-value	Post-training	Between group p-value	d-value	Within group p-value
PASAT total M (SD) APT, *N* = 31	34.4 (10.0)	0.932	49.2 (7.1)	0.502	14.8 (5.8)	<001
PASAT total M (SD) ABAT, *N* = 28	34.7 (13.8)		47.4 (11.9)*		12.8 (9.6)	<001
PASAT% Fatigability M (SD) APT, *N* = 31	–8.9 (8.3)	0.006	–4.9 (5.0)	0.890	4.0 (7.2)	0.004
PASAT% Fatigability M (SD) ABAT, *N* = 28	–3,7 (6.2)		–4.7 (6.2)*		–1.0 (8.8)	0.573
Ruff 2 & 7 ADS M (SD) APT, *N* = 31	127.8 (34.8)	0.801	135.5 (36.3)	0.812	8.1 (19.7)	0.031
Ruff 2 & 7 ADS M (SD) ABAT, *N* = 28	125.8 (23.3)		137.6 (28.6)*		13.1 (20.2)	0.002
Ruff 2 & 7 CSS M (SD) APT, *N* = 31	108.6 (27.1)	0.982	114.8 (26.4)	0.343	6.2 (13.4)	0.016
Ruff 2 & 7 CSS M (SD) ABAT, *N* = 28	108.5 (17.5)		121.0 (22.0)*		13.1 (17.2)	0.001

PASAT, Paced Auditory Serial Addition Test; ADA, Automatic Detection Speed; CSS, Controlled Search Speed; d-value, difference between pre- and post-training. * One participant missing.

There were small but significant correlations between PASAT and Ruff 2 & 7 ADS (*r* = 0.298, *p* = 0.023), though not for Ruff 2 & 7 CSS (*r* = 0.257, *p* = 0.052), and between PASAT and Digit Span (*r* = 0.341, *p* = 0.012). Also, there were small but significant correlations between PASAT-f and Ruff 2 & 7 ADS (*r* = 0.315, *p* = 0.016) and between PASAT-f and Digit Span (number of digits forward) n = 59 (*r* = 0.290, *p* = 0.033). However, we found no significant correlations between the PASAT-f variability and Ruff ADS (*r* = –0.112, *p* = 0.405), Ruff 2 & 7 CSS (*r* = –0.049, *p* = 0.715) or Digit Span (*r* = –0.241, *p* = 0.079).

No significant correlations were found between depression (HADS) and fatigability (PASAT-f) (*r* = 0.070, *p* = 0.597) or between anxiety (HADS) and fatigability (*r* = –0.076, *p* = 0.567).

### Intervention Effect

Both groups improved on the PASAT total score after training compared to baseline, indicating improved processing speed but there was no significant difference between the groups (Table 2).

**FIGURE 2:**
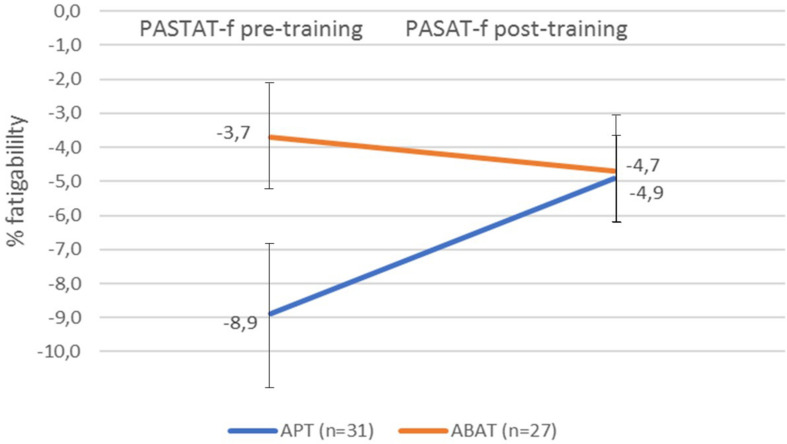
Fatigability pre-training and post-training as measured by the PASAT-f for both treatment groups. Fatigability values represent the percentage of correct answers in the first quintile of the PASAT subtracted from the number of correct answers in the last quintile of the PASAT. A negative value indicates fatigability. The error bars represent SD. APT, Attention Process Training; ABAT, Activity-Based Attention Training; PASAT-f, Paced Auditory Serial Addition Test fatigability.

There was no significant improvement in fatigability for the total group of patients (*t* = –1.579, *df* = 57, *p* = 0.120). However, as indicated in Figure 2, a significant treatment effect (d-value; *t* = –2.389, *df* = 56, *p* = 0.020) was observed, as the APT group, which started from a lower level, reduced their fatigability (PASAT f) more than the ABAT group (*p* = 0.020). Furthermore, intraindividual variability was significantly reduced in the APT-group (*t* = 2.399, *df* = 30, *p* = 0.023) but not in the ABAT-group (*t* = 1.724, *df* = 26, *p* = 0.097).

Both groups improved on Ruff 2 & 7 ADS and CSS after training (Table 2), however, no significant correlation was found between the change in fatigability pre/post intervention (d-value) and the d-values for Ruff 2 & 7 ADS (*r* = 0.129, *p* = 0.339) and CSS (*r* = 0.034, *p* = 0.802) for the total group.

There was a strong negative correlation between the baseline fatigability value and the fatigability d-value that is independent of intervention group (APT *r* = –0.801 *p* < 0.001.; ABAT *r* = –0.711 *p* < 0.001), indicating a better treatment effect in subjects with a lower baseline value, regardless of the type of attention training.

To control for the effect of the baseline fatigability value on intervention outcome, a univariate analysis of covariance was carried out with the fatigability d-value as a dependent variable, treatment as an independent variable and the baseline value of fatigability as covariate. The result showed no significant effect from the type of intervention after controlling for the baseline value of fatigability, *F* (1, 55) = 0.307, *p* = 0.581.

### *Post hoc* Analyses

To investigate the impact of ceiling effects of PASAT on the differences in the fatigability decrease observed between the groups, we counted the number of subjects who reached the maximum level (12 correct answers) in the first and last quintiles of PASAT at baseline and post-treatment. The result showed that, at baseline, 6 subjects (21%) in the ABAT group reached the ceiling in the first quintile and 1 subject (4%) in the last quintile, while in the APT group 5 subjects (16%) reached the ceiling in the first quintile and 0 in the last quintile. The Chi2 test revealed no significant differences in ceiling effect between the groups, either in the first (*Chi2* = *0.272, p* = *0.602*) or in the last quintile (*Chi2* = 2.351, *p* = 0.125).

After treatment, in the ABAT group, 12 subjects in the first quintile (43%) obtained the maximal score and 8 subjects (29%) in the last quintile. In the APT group, 20 subjects (64%) obtained the maximal score in the first quintile and 5 subjects (16%) in the last quintile. No significant differences between the groups was found for either quintile; (*Chi2* = 2.351, *p* = 0.125), (*Chi2* = 1.513, *p* = 0.219).

## Discussion

The aims of this study were to evaluate the feasibility of attention training in reducing CF after ABI, and to investigate whether targeted attention training, APT, had a better effect on CF compared to standard activity-based training (ABAT) in the subacute phase after acquired brain injury.

A significant improvement was observed for both types of cognitive training, which was measured as improved performance in the automatic and controlled speed conditions in RUFF 2 & 7, indicating a positive effect of attention training on processing speed. Furthermore, we found that CF, defined as declining performance in terms of increased number of incorrect responses on PASAT, significantly decreased after training in the APT group, but not in the ABAT group.

However, the analysis of covariance revealed that the difference in fatigability-outcome between the groups was explained by differences at baseline. These results indicate that attention training has a better effect on CF in patients with higher levels of attention dysfunction at baseline than in those with milder attention impairment. Whether APT is superior to ABAT remains unclear.

The relationship between the impairment level and the rehabilitation effect may have important clinical implications for brain injury rehabilitation. Previous studies in geriatric populations show an association between lower baseline performance in a cognitive domain and greater gains after cognitive training in that same domain (Roheger et al., 2019). On the other hand, there are studies showing higher baseline scores to be predictive of cognitive training benefits in older subjects (McKitrick et al., 1999; Fairchild et al., 2013).

A possible interpretation of the results could be that external meta-cognitive support offered by the therapist, inherent in the APT-method, might benefit the lower-level performers. An alternative explanation could be that the observed difference in the results between the groups is a mere effect of the statistical phenomenon “regression to the mean” (Bland and Altman, 1994), the initially weaker group having more room to improve than the higher performing group. Partly speaking against this is the fact that we did not find any significant differences between the groups in terms of the number of subjects that reached the ceiling of PASAT at baseline or at follow up, neither in the first nor the last quintiles of the test. However, it is undeniable that there were subjects in both groups that might have had the capacity for further improvement, as several participants reached the maximum performance level on the PASAT.

The assumption that deficiency in sustained attention and information processing speed are crucial in the development of fatigue (Kohl et al., 2009) and CF (Schwid et al., 2003) was confirmed by the significant baseline correlations between PASAT-f and Ruff 2 & 7 ADS for the total group. The correlation between Digit Span, a simple measure of working memory and attention span, and PASAT-f might further support this presumption.

Surprisingly, we found no correlation for the cognitively demanding controlled speed condition of Ruff 2&7 (CSS) with fatigability, which would have been expected from a model suggesting that CF is more sensitive to tasks demanding cognitive control than automatically executed tasks (DeLuca, 2005; Lorist et al., 2005). However, as fatigue is considered domain specific (Kluger et al., 2013), successful performance on PASAT might be more dependent on sustained attention than on selective attention. Another explanation could have been a wider performance range on the Ruff CSS measure, but that was not the case.

No baseline correlation was found between anxiety and depression and PASAT-f, which is consistent with the findings of Möller et al. (2014) and supports the notion that objectively measured CF might not be as influenced by emotional states, as self-assessed fatigue (Arnold, 2008; Möller et al., 2014). Hence, the PASAT-f measure could be suitable for an investigation of the underlying mechanisms of fatigue in brain injury that are not related to depression.

From a methodological point of view, one could question the choice of partitioning the fatigability measure, PASAT-f, into quintiles, as the narrow ranges, given the limited task length, increased the risk of ceiling effects. Previously PASAT has been divided in different ways to capture fatigability. Sometimes the performance on the first half of the PASAT has been compared with the last half (Walker et al., 2012), sometimes the first third has been compared with the last third (Morrow et al., 2015). In this study, a division into quintiles, previously applied in studies where CF has been measured with psychomotor vigilance tasks (Möller et al., 2017; Berard et al., 2019) was carried out with the purpose of making the instrument sensitive to changes between the beginning and the end. A division into halves or thirds would have given more room for improvement, but at the cost of possible sensitivity loss.

An alternative approach to the assessment of CF has been to focus on variability in performance over time, rather than mean performance decrement, where higher degrees of variability are hypothesised to be linked to dysfunctions in cognitive control mechanisms (Wang et al., 2014). In this study we did observe a correlation between reduced CF assessed with PASAT-f and reduced intraindividual variability in PASAT. The measures are interdependent though, preventing firm conclusions from being drawn, and it is noteworthy that the variability did not correlate with the independent attention measures (Ruff 2 & 7 and Digit Span). Response time variability as a measure of CF has not been much used in studies on patients with stroke or TBI, as opposed to decrement-measures. However, this approach is of particular interest, since response-time variability, in contrast with performance-decline measures, has been shown to significantly correlate with subjectively reported fatigue in patients with MS (Bruce et al., 2010), and also in patients with mild TBI (Möller et al., 2017). In a future study it would be interesting to investigate the correlation between variability in performance and performance decline in brain injured patients more closely and to unravel whether variability in performance might be more closely related to subjective fatigue experience than objectively measured performance decrement.

### Limitations

The study has some methodological weaknesses, apart from the issue regarding the principles for partitioning the PASAT-f discussed above. Due to the fact that the data was collected from a clinical trial not targeting CF (Bartfai et al., 2014), CF was not an inclusion criterion for participation. However, as CF is a cardinal symptom after ABI, we assumed that the randomisation will ensure an equal distribution in both groups. A preselection of patients with CF would have reduced the baseline difference between the intervention groups, thereby making comparisons of the results of the interventions for CF clearer and more convincing.

Secondly, the influence of spontaneous recovery on training effects in the early stage after ABI could be regarded as a limitation. However, both groups were in the same stage of recovery and, thus, those effects could be assumed to be similar. This problem could have been remedied by including a control group receiving no treatment, however, ethical issues preclude withholding treatment when it is available.

The interpretation of the PASAT results is slightly problematic. An initial practice effect has earlier been demonstrated (Tombaugh, 2006) with repeated administration. Therefore, we cannot rule out that some of the improvement was related to a practice effect.

Also, neither the patients nor the therapists were blinded to the type of intervention, which might have affected the results through placebo effects, even though different therapists were in charge of treatment and assessment. Lastly, it should be noted that the strict enrolment criteria used in this study (Markovic et al., 2017) demand caution in the generalisation of the results.

## Conclusion

The advantage of this randomised controlled study is that it addresses cognitive training as a possible method for reducing CF – an area where studies are currently lacking. It also suggests that it might be feasible to reduce CF through attention training in patients with acquired brain injury. It can, therefore, inspire future studies, where objective measures are used as a complement to self-assessment scales to measure fatigue. The study also indicates that patients with high levels of CF might improve more from attention training than patients who have less severe CF. Whether structured or activity-based attention training is provided appears to be less important. Due to methodological drawbacks the results are tentative and future studies are required to confirm the validity of the findings. Such studies should include only patients exhibiting CF and the results of the intervention groups should be compared with the result of a control group receiving no attention training.

## Data Availability Statement

The raw data supporting the conclusions of this article will be made available by the authors, without undue reservation.

## Ethics Statement

The studies involving human participants were reviewed and approved by the Ethics Review Board Stockholm (2007/1363-31 and 2012/3: 4). The patients/participants provided their written informed consent to participate in this study.

## Author Contributions

AB and GM were responsible for study design. GM implemented the research. MM conceptualized the fatigability measure and did the statistical analyses and prepared the figures. AH was the first author and did the manuscript draft. All authors contributed to the manuscript.

## Conflict of Interest

The authors declare that the research was conducted in the absence of any commercial or financial relationships that could be construed as a potential conflict of interest.
